# Prostate Cancer Patients–Negative Biopsy Controls Discrimination by Untargeted Metabolomics Analysis of Urine by LC-QTOF: Upstream Information on Other Omics

**DOI:** 10.1038/srep38243

**Published:** 2016-12-02

**Authors:** M. A. Fernández-Peralbo, E. Gómez-Gómez, M. Calderón-Santiago, J. Carrasco-Valiente, J. Ruiz-García, M. J. Requena-Tapia, M. D. Luque de Castro, F. Priego-Capote

**Affiliations:** 1 Department of Analytical Chemistry, Annex Marie Curie Building, Campus of Rabanales, University of Córdoba, E-14071, Córdoba, Spain; 2 Institute of Biomedical Research Maimónides (IMIBIC), Reina Sofía University Hospital, University of Córdoba, E-14004, Córdoba, Spain; 3 Department of Urology (IMIBIC), Reina Sofia University Hospital, University of Córdoba, E-14004, Córdoba, Spain

## Abstract

The existing clinical biomarkers for prostate cancer (PCa) diagnosis are far from ideal (*e.g.,* the prostate specific antigen (PSA) serum level suffers from lack of specificity, providing frequent false positives leading to over-diagnosis). A key step in the search for minimum invasive tests to complement or replace PSA should be supported on the changes experienced by the biochemical pathways in PCa patients as compared to negative biopsy control individuals. In this research a comprehensive global analysis by LC–QTOF was applied to urine from 62 patients with a clinically significant PCa and 42 healthy individuals, both groups confirmed by biopsy. An unpaired *t*-test (*p*-value < 0.05) provided 28 significant metabolites tentatively identified in urine, used to develop a partial least squares discriminant analysis (PLS-DA) model characterized by 88.4 and 92.9% of sensitivity and specificity, respectively. Among the 28 significant metabolites 27 were present at lower concentrations in PCa patients than in control individuals, while only one reported higher concentrations in PCa patients. The connection among the biochemical pathways in which they are involved (DNA methylation, epigenetic marks on histones and RNA cap methylation) could explain the concentration changes with PCa and supports, once again, the role of metabolomics in upstream processes.

Despite prostate cancer (PCa) is a slow growing tumor mainly affecting old men, it is the second most frequently occurring cancer and the third leading cause of cancer death in men^1^. The main limiting factor in the diagnosis of PCa is the absence of symptoms at early stages. Despite the pathogenesis of PCa has not been completely elucidated, major risk factors associated to its development include age, race, and occurrence of PCa in the family line^2^. Likewise, lifestyle, diet or environmental factors can induce genetic modifications and initiate carcinogenesis processes^3^.

To date few biomarkers have reached clinical use since the proposed candidates need to be specific for PCa to cause no alteration or expression in other human tissues or diseases. Urinary biomarkers can be broadly classified into DNA, RNA, protein and, recently, metabolite-based markers. Currently, prostate specific antigen (PSA) is the marker of choice for PCa screening. However, it is still not able to distinguish clinically relevant tumors from indolent ones and PSA use in screening is being downgrading. Various PSA-related derivatives, such as PSA velocity, PSA density, and free-to-total PSA ratio, have provided only limited improvements in terms of specificity^4^. RNA urine markers, the most clinically developed to date, include prostate cancer antigen 3 (PCA3), and a fusion of the 50-untranslated region of androgen-regulated transmembrane-serine protease gene (TMPRSS2) with virus E26 oncogene (ERG) (TMPRSS2:ERG) among the most used^5^. Recently, the Food and Drug Administration has approved PCA3 for its ability to predict PCa in patients with elevated PSA and a negative biopsy, but they are still considered at risk and may require a repeated biopsy. Furthermore, other studies have concluded that PCA3 has limited value in predicting aggressive cancers^6^. Similarly, although the TMPRSS2:ERG fusion was useful as a screening tool, its prognostic utility is controversial^7^.

As stated above, genetic alterations caused by epigenetic factors can also play a significant role on PCa occurrence^8^. Increased methylation of cytosine at CpG islands of the glutathione S-transferase Pi 1 (GSTP1) promoter has shown to be a very common epigenetic alteration in PCa, which has been well characterized in tissues and seems to be very promising for urine biomarker development^9^. Furthemore, aberrations in post-translational modifications (PTMs) of histones have shown to occur in PCa cells, in particular acetylation and methylation of specific lysine and arginine residues are being investigated as predictor of the risk of PCa recurrence^10^. Regarding to metabolite markers, sarcosine (N-methylglycine) is one of the main candidates found to be associated with PCa progression to metastasis, with a significant predictive value for PCa detection in urine samples^11^. However, several studies reveal that the role of sarcosine as urinary biomarker is still controversial and further validation studies are warranted^12^. Some metabolites related to genetic alterations in PCa have been found altered in biological samples from PCa patients. For instance, Jung *et al*. found 7-methylguanine up-regulated in malignant tissue from PCa individuals versus non-malignant tissue^13^; while both methylguanosine and dimethylguanosine appeared down-regulated in urine from PCa patients^14^.

So far, the lack of conclusive studies makes urgently needed alternative screening tools based on no or minimum invasive tests applied to biological samples such as blood or urine; tools that allow discrimination of individuals with clinically significant PCa from those with no disease. Recent advances in analytical instrumentation have facilitated the present development of metabolomics. Since metabolite profiles closely reflect the total cellular state, metabolomics has been developed into a more integrative approach in comparison with other omics approaches.

Urine was chosen as biological material for development of the present research due to its non-invasiveness, easy collection and transportation, stability during short and long-term storage and, as the most important, the presence of end-products from cancer metabolism released either into the urine or carried within prostatic cells and directly released into the urethra through prostatic ducts. All these aspects suggest urine as an ideal biofluid for PCa studies; therefore, comprehensive analysis of urine metabolome by LC–MS/MS in high-resolution mode was applied to search for discrimination between patients with clinically significant PCa and healthy individuals, both groups confirmed after biopsy. The set of significant metabolites tentatively identified was used to study the metabolic pathways potentially involved in PCa.

## Methods

### Ethical Statement

The selected individuals were part of a cohort of patients scheduled for prostate biopsy according to the European Association of Urology (EAU) guidelines^15^. All members of the cohort signed the informed consent before involvement in the project. The experiment was planned following the guidelines dictated by the World Medical Association Declaration of Helsinki (2004), and was supervised by the ethical review board of Reina Sofia University Hospital (Córdoba, Spain) that approved the experiments.

### Subjects

Morning urine samples were collected in the Urology Department of the Reina Sofia Hospital from 104 patients on the same day of prostate biopsy before the intervention. First morning urine was discarded for development of this research. All individuals were treated with the same dose of ciprofloxacin the day before sampling, which was carried out after overnight fasting of at least for 12 h. The samples were aliquoted in 1.5 mL tubes and stored at −80 °C until analysis. The individuals were grouped in two categories, PCa patients and negative biopsy controls, as Table 1 shows. The PCa category included 62 patients with clinically significant PCa confirmed by prostate biopsy. Clinically significant PCa had to meet at least one of these criteria: clinical stage >T1c and/or Gleason pattern >6 and/or more than 3 positive cores and/or >50% cancer per core. On the other hand, no cancer was detected by histological analysis of the control group, which included 42 individuals. 36 out of 62 (58%) PCa patients showed a positive digital rectal examination (DRE) in contrast to negative biopsy controls who gave negative DRE. The mean age of patients with PCa was 71 versus 62 years of the control group. Additionally, no significant differences were found in terms of prostate volume between the two groups, as well as in+ the number of biopsies.

Two PSA measurements were carried out to each patient: one before the biopsy, as a decision tool to undergone prostate biopsy, and a second one on the same day of the biopsy. Logically, as shown in Table 1, PCa patients provided higher PSA in both measures. In the first test, all PCa individuals gave values above 3 ng/mL, with 27 cases between 3 and 10 ng/mL and 35 patients above 10 ng/mL. These values were quite similar in the second PSA test.

On the other hand, the negative biopsy control included 39 patients (92.9%) with serum PSA above 3 ng/mL. However, this group was clearly dominated by individuals with PSA levels between 3 and 10 ng/mL. A clear change was observed in the second PSA test since most of the control group individuals gave serum PSA levels below 3 ng/mL in the measurement of the day of biopsy.

### Reagents

Acetonitrile (ACN) (LC–MS optimum grade) from Fisher Scientific (Fair Lawn, NJ, USA) and deionized water from a Millipore Milli-Q water purification system (Millipore, Bedford, MA, USA) were used to prepare the chromatographic mobile phases. LC–MS grade formic acid from Scharlab (Barcelona, Spain) was used as ionization agent in LC–MS/MS analysis. Ammonium formate from Fluka (Sigma–Aldrich, St. Louis, USA) was used to adjust the pH of urine.

### Apparatus and Instruments

Centrifugation was carried out by a Thermo Sorvall Legend Micro 21R thermostated centrifuge (Thermo Fisher Scientific, Bremen, Germany). The urine samples were analyzed by an Agilent 1200 Series LC system hyphenated to an Agilent 6540 QTOF with a dual electrospray ionization (ESI) source (Agilent Technologies, Santa Clara, CA, USA). Agilent MassHunter Workstation (version B05.01) was the software for data acquisition.

### Sample Preparation

After thawing at room temperature, urine samples were vortex-mixed for 1 min and centrifuged at 21000 ×* g* for 5 min. Then, 50 μL of the supernatant were 1:2 (v/v) diluted with 5 mM ammonium formate in water (pH 5.5–7.5) prior to LC–MS/MS analysis.

### LC–QTOF Analysis

Chromatographic separation was performed by using a Mediterranea Sea C18 analytical column (50 × 4.6 mm i.d., 3 μm particle size) from Teknokroma (Barcelona, Spain), thermostated at 25 °C. The initial mobile phase was a mixture of 98% phase A (0.1% formic acid in water) and 2% phase B (0.1% formic acid in ACN). After injection, the initial mobile phase was kept under isocratic conditions for 1 min; then, a linear gradient of phase B from 2% to 100% was applied within 16 min. The flow rate was 0.6 mL/min during the chromatographic step. The total analysis time was 17 min, and 5 min were required to re-establish the initial conditions. The injected volume was 5 μL in each ionization mode and the samples were randomly run. The injector needle was washed 5 times with 20:80 ACN–water between injections and the needle seat back was flushed with 20:80 ACN–water at 4 mL/min for 10s to avoid cross contamination. The autosampler was kept at 4 °C to increase sample stability. Urine samples from several healthy volunteers were collected, mixed to obtain a pool, aliquoted and stored as quality control (QC) samples to be daily analyzed at the beginning of the sequence and after every 8 injections to ascertain that the mass spectrometer performance was stable during the analysis of the samples set. Thus, 2 QCs were run per day following the same protocol as that used for samples.

The settings of the electrospray ionization source, operated in the negative and positive ionization modes, were as follows: capillary voltage ± 3.5 kV, fragmentor voltage 130 V, N_2_ pressure in the nebulizer 40psi, N_2_ flow rate and temperature as drying gas 12 L/min and 325 °C, respectively. The instrument was calibrated and tuned according to procedures recommended by the manufacturer. Each sample was analyzed in triplicate by setting data acquisition in centroid mode at 2 spectra per second in MS mode and 1 spectrum per second in MS^2^ mode in the extended dynamic range mode (2 GHz). A gas-phase fractionation approach was used to increase the identification capability^16^. For this purpose, each of the three replicates from each sample was acquired with different ranges of precursor selection for MS/MS detection. In all cases, the acquisition range in MS scanning mode was from 60 to 1200 *m/z*. The maximum number of precursors selected per cycle for MS/MS fragmentation was set at 2, with an exclusion window of 0.25 min after two consecutive selections of the same precursor ion. Criteria for selection of precursor ions were established for each of the three runs per sample to obtain the maximum spectral information. Thus, in both ionization modes the mass range for precursor selection in the first run was set at 60–250 *m/z* with 10 eV as collision energy. The second and third runs involved precursor selection in the range 250–450 and 450–1200 *m/z* with collision energies of 20 and 30 eV, respectively. The instrument gave typical resolution 15000 FWHM (Full Width at Half Maximum) at *m/z* 118.0863 and 30000 FWHM at *m/z* 922.0098. To assure the desired mass accuracy of the recorded ions, continuous internal calibration was performed during analyses by using signals at *m/z* 121.0508 (protonated purine) and *m/z* 922.0097 [protonated hexakis (1 H, 1 H, 3H- tetrafluoropropoxy) phosphazine or HP-921] in positive ionization mode. In negative ionization mode, ions with *m/z* 119.0363 (purine anion) and *m/z* 1033.9881 (adduct of HP-921) were used.

### Data Processing and Statistical Analysis

Raw data files were converted to mzData files using Mass Hunter Workstation software (version B.07.00 Qualitative Analysis, Agilent Technologies, Santa Clara, CA, USA); then, the data from each polarity were separately processed in R statistical language (version 3.1.3, http://www.r-project.org/) using the open-free XCMS (version 1.44.0)^17^ and CAMERA (version1.24.1) R-packages. The XCMS package was used for processing mzData files by extracting potential molecular features (MFs) considering only ions exceeding 1500 counts peak height with a peak width between 10 and 60s, a signal-to-noise threshold of 10 and 10 ppm of error in mass accuracy. Only molecular entities detected in at least 75% of the samples belonging to at least one of the groups under study (PCa and control individuals) were considered. Then, alignment of retention times across samples, grouping and integration were executed using the obiwarp method from the “retcor” function. In the next step, the CAMERA package was used to correlate potential precursor ions as [M–H]^−^, [M + Cl]^−^ and [M + HCOO]^−^ in negative ionization mode, and as [M + H]^+^ and [M + Na]^+^ in positive ionization mode, as well as isotopic peaks from the same molecular entity. Ions with identical elution profiles and related *m/z* values (representing different adducts or isotopes of the same compound) were grouped as a unique feature to remove redundant information. This process resulted in a data set for each polarity mode containing the peak area values in the apex of chromatographic peaks for all molecular entities characterized by accurate mass and retention time (RT). As the samples were injected in triplicate, the average area of the three injections was calculated for each potential molecular feature.

Normalization of the data set in each polarity was based on the mass spectrometry total useful signal (MSTUS) method that attempts to limit the contributions of xenobiotics and endogenous substances to the normalization factor by including only peaks present in all samples^18^. Thus, this normalization takes into account the differences in volume of urine sampled per individual since metabolites would be more concentrated and give higher signal when the volume of collected urine is lower.

The data matrix from each polarity was introduced as *.csv* format into the Mass Profiler Professional (MPP) software (version 13.1, Agilent Technologies, Santa Clara, CA, USA) for statistical analysis. A supervised analysis of the data set was done by partial least squares discriminant analysis (PLS-DA). Additionally, an unpaired *t*-test with Benjamini-Hochberg false discovery rate (FDR) was executed to find metabolites significantly different between the two groups of patients, considering a *p*-value threshold of 0.05. Identification of the most relevant entities was supported on MS and MS/MS information by searching in the METLIN MS and MS/MS database (http://metlin.scripps.edu) and the Human Metabolome Database (HMDB, v. 3.6). An accuracy error of 5 ppm was set both in MS and MS/MS data to confirm the tentative identification of metabolites.

Normalized data were uploaded at MetaboAnalyst 3.0, a web-tool based on analytical high-throughput metabolomics studies (http://www.metaboanalyst.ca) for metabolomic data analysis and interpretation.

## Results and Discussion

### Data Pretreatment and Exploratory Analysis

Normalization of urine is a critical step in metabolomics analysis owing to variability of volume excretion among individuals. Under normal conditions, urinary creatinine excretion is relatively constant and easily measurable; therefore, it is a common practice to normalize urinary metabolite levels to the response of this endogenous metabolite. However, creatinine production may vary by an external stressor such as kidney impairment^19^. For this reason, alternatives such as the so-called “MSTUS” normalization method are gaining popularity. This method has been previously compared to other common normalization strategies such as urine volume, osmolarity and creatinine concentration, concluding that it reduces the external variability among biological replicates^18^. For this reason, MSTUS was selected for normalization of urine samples. Thus, the peak area of each detected compound was divided by the sum of peak areas of all MFs detected in each sample. The MSTUS approach was preliminarily applied to a data set generated by analysis of QC samples injected along the batch for estimation of the methodological variability in the detection of MFs. As a result, the within-laboratory reproducibility, expressed as relative standard deviation (RSD), ranged from 3.15 to 16.9%. These values indicated that the method was reproducible enough for metabolomics research and no QCs correction within days was required.

The number of MFs extracted by the XCMS R-package from the data obtained after analysis of the cohort in negative and positive ionization modes was 1127 and 781, respectively. These results were obtained after removing redundant information from adducts and isotopes provided by the CAMERA R-package, and considering only MFs present in at least 75% of the samples pertaining to one of the groups (healthy individuals or PCa patients).

### Tentative Identification of Significant Entities Detected by MS/MS

An unpaired *t*-test was applied to identify statistically significant differences (*p* < 0.05), in terms of concentration, between the two groups under comparison, using the Benjamini-Hochberg FDR to avoid false positive. The statistical test reported 111 and 75 statistically significant entities in the data sets obtained in positive and negative ionization modes, respectively. Among them, 28 metabolites were tentatively identified by MS/MS in this research according to the requirements previously established. The rest of significant MFs were not identified owing to either bad quality of the MS/MS spectra or lack of information included in most databases; therefore, only compounds with high quality MS/MS spectra were considered in this step. Table 2 lists the tentatively identified metabolites, with special emphasis on amino acids and derivatives, which represents 78.6% of all them. The list also included sulfonic acids (10.7%) and nitrogenous bases derivatives (10.7%). Moreover, it should be mentioned that 28.6% of the metabolites were acetylated compounds such as acetylcitrulline, acetylarginine, acetylputrescine or acetyllysine, while 25% of them represented methylated amino acids or nitrogenous bases as methylguanosine, methyllysine, methylhistidine or dimethylarginine. Information on identification in terms of MS precursor ion, the most representative product ions, RT, formula, *p*-value, corrected *p*-value and regulation in PCa compared to control individuals is also included. Several of these metabolites were detected in both positive and negative ionization polarities, but only the ionization mode providing the highest sensitivity was considered.

Supplementary Table 1 shows the reproducibility values for each identified metabolite, expressed as RSD. The values have been calculated prior to and after MSTUS normalization in order to evaluate the correction effect. As can be seen, RSD was reduced in all cases after application of the MSTUS normalization strategy.

### Partial Least Squares Discriminant Analysis (PLS-DA)

**Figure 1:**
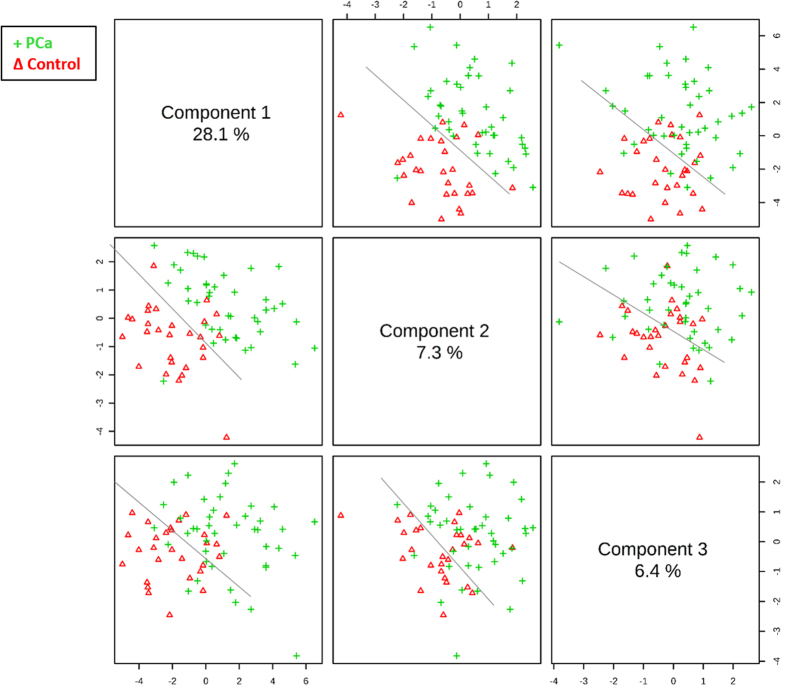
PLS-DA scores plot for discrimination of PCa and negative biopsy patients by urine metabolomics analysis. PLS models were built with 70% of samples randomly selected from each group.

As commented above, one of the main limitations concerning PCa diagnosis is the stage at which the disease is typically detected and the characteristics of the existing available tests. Therefore, new tests with low false negative and positive rates are demanded for preliminary PCa screening. In this study, a discrimination model based on PLS-DA was built using a new data set exclusively constituted by the tentatively identified significant metabolites. The resultant PLS-DA model showed a clear separation between patients with PCa and control individuals, as the scores plot in Fig. 1 illustrates.

The combination of principal component 1 (PC1) and PC2 provides the greatest discrimination between the two classes: PCa and healthy individuals, which are separated through the diagonal between components in Fig. 1. The values of sensitivity and specificity obtained in the training model considering 70% of the samples randomly selected were 88.4% and 92.9%, respectively. Then, the stability of the model was assessed by external validation using 30% of the samples excluded in the training step. Validation reported sensitivity and specificity of 63.2% and 78.6%, respectively. Table 3 summarizes this information and includes the percentage of positive and negative predictive values.

**Figure 2:**
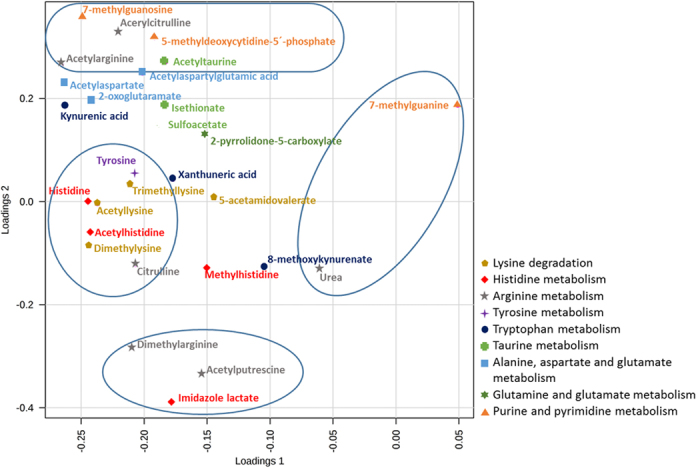
PLS-DA loadings plot to discriminate patterns of PCa and negative biopsy individuals.

Since the combination of PC1 and PC2 led to the best discrimination between the two groups, these principal components were selected to evaluate the loadings plot corresponding to the PLS-DA, shown in Fig. 2.

Thus, through PC1, which explains 28.1% of the variability, several metabolites such as urea and 7-methylguanine contributed to the clustering of the PCa patients; while amino acids such as tyrosine, citrulline and histidine together with acetylated and methylated amino acids such as acetyllysine, acetylhistidine, dimethyllysine and trimethyllysine were relevant to discriminate the control group of individuals with negative biopsy. On the other hand, and through PC2, imidazole lactate, acetylputrescine and dimethylarginine contributed to characterize the healthy group, while a heterogeneous group of metabolites including 5-methyldeoxycytidine-5-phosphate, 7-methylguanosine, acetylcitrulline, acetylaspartatylglutamic acid and acetyltaurine supported the differentiation of the PCa patients group.

### Biological Interpretation According to the Obtained Results

After identification of metabolites found at significantly different concentrations between control and PCa individuals, they were studied and classified according to their connection across the biochemical pathways in which they are involved, and grouping them according to their involvement in the pathways. As can be seen in Table 2, 27 significant metabolites were present at lower concentration in PCa patients than in control individuals, while only one reported higher concentration in PCa patients. All these metabolites have been grouped in the table according to the metabolic pathway in which they are preferentially involved. As can be seen, the metabolism of amino acids was predominantly altered by the occurrence of prostate tumor. In fact, the concentration of 3 amino acids and 19 related metabolites, with special emphasis on acetylated and methylated amino acids, were altered in PCa individuals. The main pathways involving metabolism of amino acids altered by prostate tumor occurrence affected lysine, histidine, arginine, alanine, aspartate and glutamate, as well as aromatic amino acids, particularly tryptophan and tyrosine.

On the other hand, 3 purines and pyrimidines derivatives were also altered in cancer. Concretely, one of them —7-methylguanine— was the exclusive significant metabolite down regulated in PCa, as Table 2 shows.

According to the list of significant metabolites, three biological processes can be mainly emphasized to be discussed in this research: DNA methylation, epigenetic marks on histones and RNA cap methylation. The three biological processes are independently discussed to explain the potential reason for the metabolic discrimination detected in human urine from PCa patients and negative biopsy individuals.

#### Methylated and Acetylated Amino Acids Related to Histone Protein Modifications

Histone modifications together with DNA methylation are well-recognized epigenetic mechanisms of gene transcriptional regulation and play essential roles in PCa tumor initiation and progression. The N-terminal tails of histones, peripherally positioned around the nucleosome core, are subjected to various covalent post-translational modifications (PTMs) such as acetylation, methylation, phosphorylation, citrullination and ubiquitination, among others, by specific chromatin-modifying enzymes. The pattern of these modifications has been referred to as the “histone code” and determines whether the chromatin will adopt a transcriptionally active or inactive state^20^. Acetylation and methylation are widely considered the two most important PTMs occurring in histones. Acetylation induces an open chromatin conformation to regulate the accessibility of transcription factors by blocking the normal electrostatic interaction between positively charged N-terminal basic residues such as lysine, arginine and histidine located at tails of the histone, and negatively charged DNA phosphate groups. Acetylation has shown to alter the structural core of nucleosomes and chromatin, enhancing gene expression as well as DNA reparation and cytokine-activated signal transduction^21^,^22^. The level of acetylation is balanced by two classes of enzymes: histone acetyl transferases (HATs) and histone deacetylases (HDACs). Aberrant activities of these enzymes such as excessive recruitment of histone deacetylases and mutation in acetyltransferases have been implicated in silencing of key oncosuppressor genes identified as an early event of oncogene activation to cancer pathways^23^.

Methylation is based on the same biochemical mechanism and, therefore, also contribute to modify chromatin conformation. Histone methylation is characterized by a dynamic mark in health and inheritance. By analogy to acetylation, the enzymes involved in these reversible marks include histone methyltransferases (HMTs) and histone demethylases (HDMs). However, in contrast with acetylation, methylation of specific lysine or arginine residues can result in transcriptional activation or repression for gene silencing. Additionally, combined methylation marks can present different roles to the same marks appearing in isolation.

Inappropriate targeting of histone modifying enzymes is often responsible for aberrant histone modifications. Thus, abnormal modifications in the profile of histone acetylation and methylation have been found in PCa patients, which has opened a new group of promising biomarkers with diagnostic potential to predict disease severity. As previously emphasized, these epigenetic alterations are interestingly reversible by enzymatic action. Thus, identifying key epigenetic pathways in cancer cells might pave the way to innovative therapeutic approaches^24^.

**Figure 3:**
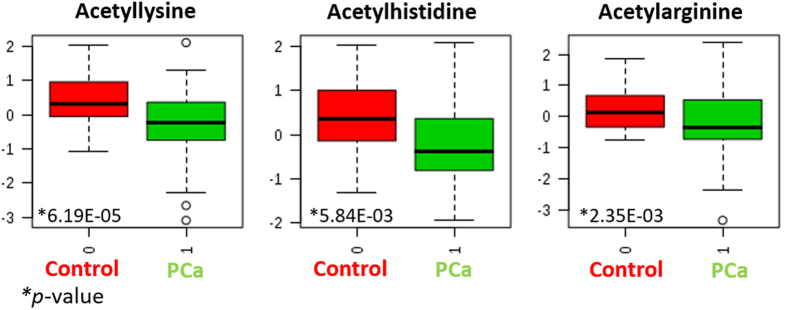
Box and whisker plots from the three acetylated basic residues–lysine, arginine and histidine– in the two evaluated groups.

In the present research, the most interesting result came from the levels of acetylated and methylated basic amino acid residues by application of a metabolomics workflow for urine analysis. Particularly, acetylated and methylated lysine, arginine and histidine residues were among the most statistically significant metabolites contributing to explain the differences in urine composition between PCa patients and healthy individuals. Acetyllysine, acetylarginine and acetylhistidine were found at high-significantly lower concentration in urine from PCa patients. Figure 3 shows the box-and-whisker plots for these three acetylated basic residues in the two evaluated groups.

The fold change values considering control individuals versus PCa patients were 1.33, 1.34 and 1.26 for acetylated lysine, arginine and histidine, respectively. This result strongly agrees with expression levels of histone acetylases (HACs), HDACs and HATs in PCa patients. Thus, HDACs are often overexpressed in prostate cancer associated to reduced histone acetylation^25^, responsible for tumor suppressor gene silencing and malignant transformation. Sato *et al*. proved that the inhibition of histone deacetylase efficiently suppressed cell growth of three prostate cancer lines together with down-regulation of the androgen receptor, regardless of their hormone sensitivity, based on miRNA-mediated suppression^26^.

Citrullination is an additional PTM occurring in histones, which involves the enzymatic conversion (by peptidylarginine deiminase 4, PADI4) of peptidyl arginine to citrulline to neutralize the positive charge of the former. Different studies have pointed out a transcriptional repression role to citrullination through connection to deacetylation^27^, although this PTM also seems to facilitate gene expression in early embryos by creating a platform for HAT assembly, thus leading to the enhancement of histone acetylation^28^. At the metabolite level, the present study reports that both citrulline and acetylcitrulline were significantly at higher concentrations in healthy individuals as compared to PCa patients. Therefore, both citrullination and acetylation of citrulline reported a behavior similar to that experienced by acetylated arginine. Citrullination could also contribute to explain the metabolic differences in urine from PCa patients as compared with that from negative biopsy individuals. Supplementary Figure 1 shows the box and whisker plots comparing the concentration levels of citrulline and acetylcitrulline found in urine from PCa patients versus that from healthy individuals.

**Figure 4:**
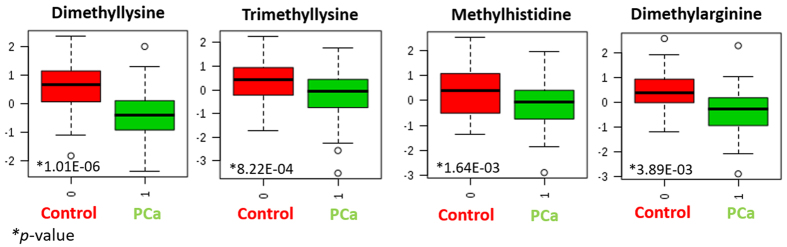
Box and whisker plots for methylated residues to help in interpreting the role of histone methylation in PCa.

Similar to acetylation, several evidences of histone methylation marks were detected as significant metabolites by comparing PCa and healthy individuals. Thus, dimethyl and trimethyllysine, dimethylarginine and methylhistidine were in the list of significant metabolites. Among them, it is worth mentioning the role of dimethyllysine, the most significant compound in terms of *p*-value, that also experienced the highest concentration change when comparing controls and PCa patients (in fact, the fold change for this metabolite was 1.74). Figure 4 shows the box and whisker plots for methylated residues that could aid to interpret the role of histone methylation in PCa.

Concerning methylation, the lower levels found for methylated residues in PCa patients would reveal a higher demethylation in this group of individuals leading to a higher gene silencing effect. Furthermore, several studies have suggested that decreased histone acetylation and methylation may be a consequence of cancer cell metabolism, since a rapid proliferation of cells and a higher activation of macromolecular biosynthesis may deplete the levels of acetyl and methyl donors such as acetyl coenzyme A and S-adenosyl methionine (SAM)^29^.

On the other hand, three metabolic products of lysine, arginine and histidine significantly detected in this study were 5-acetamidovalerate, acetylputrescine and imidizol lactate, respectively. All these metabolites were present at higher concentrations in negative biopsy patients as compared to PCa cases (see Supplementary Figure 2), thus also indicating a higher activation of basic amino acids metabolism.

#### DNA Methylation

DNA methylation occurs when a methyl group is attached to the fifth carbon of cytosine nucleotide in a process catalyzed by DNA methyltransferases with SAM as methyl donor. The two most common mechanisms of aberrant methylation are global hypomethylation and site specific hypermethylation. Both processes impact gene expression, genome stability, genetic imprinting and cellular differentiation. DNA methylation can be enzymatically removed by several mechanisms including base excision repair, nucleotide excision repair and hydrolysis^30^,^31^.

In mammalians, 5-methylcytosine (5mC) is sequentially oxidized into 5-hydroxymethylcytosine (5hmC), further to 5-formylcytosine (5fC) and 5-carboxylcytosine (5caC)^32^,^33^. These three oxidized methylcytosines point out the occurrence of both passive and active DNA demethylation and also serve as stable epigenetic marks^34^. Enzymatic mechanisms for removal of DNA methylation may release repaired products into the bloodstream and consequently they can appear in the urine. In fact, the deoxynucleosides of 5mC and 5hmC –namely 5-methyl-2-deoxycytidine (5mdC) and 5-hydroxymethyl-2-deoxycytidine (5hmdC)– have recently been detected and quantified in urine^35^,^36^, and several biological processes such as apoptosis can degrade DNA into single deoxynucleosides^37^. According to these studies, urinary 5mdC and 5hmdC contents may offer a view of the metabolic and functional status of tissues or organs in the human body.

**Figure 5:**
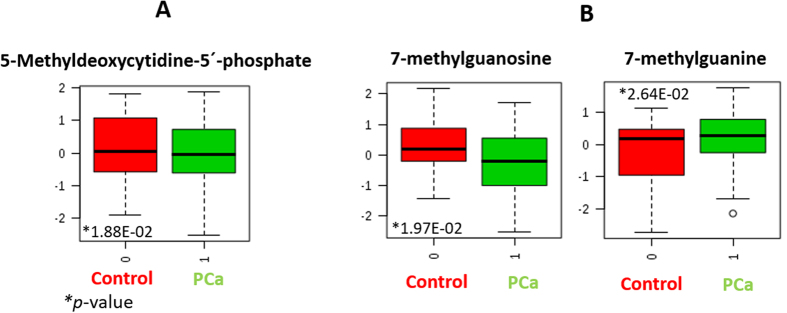
Box and whisker plots comparing the concentration of (A) 5-methyldeoxycytidine-5′phosphate and (B) 7-methylguanosine and 7-methylguanine, detected in urine from PCa patients and negative biopsy individuals.

In this research 5mdC phosphate has been significantly detected at different concentration in PCa patients as compared to negative biopsy individuals. Particularly, its concentration was lower in PCa cases, as Fig. 5A shows.

This result could be interpreted by a lack of efficiency in the machinery for repairing DNA methylation. As a result, this 5mdC epigenetic mark would be less metabolized and, therefore, detected at lower concentration level in urine from PCa patients. This finding is consistent with that reported by Morey *et al*., who found genomic DNA hypomethylation and loss of 5-methyl-2-deoxycytidine in both early and late stages of mouse PCa adenocarcinoma^38^.

Increased evidences also connect DNA methylation and methylated DNA lesions. Previous studies have reported that SAM was able to methylate DNA (O-methylguanine, 7-methylguanine and 3-methyladenine) without enzymatic involvement to produce promutagenic and procarcinogenic lesions^39^,^40^. This assumption could also explain the higher levels of 7-methylguanine in urine from PCa patients as compared to negative biopsy controls. In fact, previous studies have found elevated levels of methylguanine in serum and urine from cancer patients. For instance, Jung *et al*. found a significant increase of 7-methylguanine in malignant tissue from PCa patients as compared to non-malignant tissue^13^.

#### RNA cap Methylation

Eukaryotic mRNA is modified by the insertion of 7-methylguanosine ‘cap’ to the first transcribed nucleotide. This modification is required for efficient gene expression to produce proteins and cell viability as the 7-methylguanosine cap allows translation of the majority of mRNAs, stabilizes mRNA against exonucleases and promotes transcription, splicing, polyadenylation and nuclear export of mRNA.

In the present research, 7-methylguanosine was found at significantly different concentration in PCa patients versus negative biopsy individuals. Particularly, this metabolite was found at lower concentration in PCa patients, which suggests that the translation process would be impeded in patients with the tumor.

The metabolite resulting from potential depurination of 7-methylguanosine (7-methylguanine) was also significantly different in the comparison of PCa patients and healthy individuals. Particularly, the concentration profile of 7-methylguanine was inverse to that described for 7-methylguanosine. Thus, 7-methylguanine was detected at higher concentration in PCa individuals than in healthy cases. Figure 5B shows the box-and-whisker plots comparing the concentration of 7-methylguanosine and 7-methylguanine detected in urine from PCa patients and negative biopsy individuals. Depurination could explain the reduced concentration of 7-methylguanosine in PCa patients. This statement is supported on the concept of “decapping”, the major mechanism by which the mRNA cap methylation is reversed by removing the entire 7-methylguanosine cap. This process would cause serious errors during genes translation, with cell apoptosis as a result.

#### Miscellaneous

Tryptophan metabolism has been widely related both to cancer and immune system. In fact, kynurenic acid has been proposed as a biomarker in urine sediment collected from subjects following a DRE to discriminate PCa patients from healthy subjects. In the present study, kynurenic acid, xanthunerate and 8-methoxykynurenate, involved in tryptophan metabolism, presented lower levels in PCa individuals as compared to controls.

Other group of metabolites significantly decreased in urine from PCa patients in comparison to the control group included acetyltaurine, isethionate and sulfoacetate. These three metabolites, excreted in urine, are end-products from the metabolism of taurine. Chatzakos *et al*. showed that N-acyltaurines are anti-proliferative in PCa cells^41^. Also, Tang *et al*. investigated the anti-prostate cancer metastasis effect of taurine, and they proved that taurine attenuated PSA and several metastasis-related genes in human PCa cells, such as LNCaP and PC-3. In addition, taurine inhibited migration of LNCaP and PC-3^42^; therefore, it seems that taurine metabolism could be crucial in PCa regulation.

## Conclusions

In this research, a comprehensive global analysis by LC–QTOF of urine from PCa patients and negative biopsy control individuals has allowed discrimination between both groups. An unpaired *t*-test (*p*-value < 0.05) provided 28 significant metabolites involved in biological processes such as the regulation of epigenetic marks, essentially DNA methylation and modifications of histones, as well as RNA cap methylation. A PLS-DA model allowed discriminating both groups of individuals with sensitivity and specificity values of 88.4% and 92.9%, respectively.

This study revealed changes at metabolomics level that can be associated with epigenetic marks widely studied as promising markers for diagnostic of PCa due to their involvement in gene expression or silencing. An additional process such as RNA cap methylation also reported significant differences between PCa patients and controls, which emphasizes the relevance of RNA transcription. Therefore, the analysis of urine revealed metabolite changes that can be interpreted according to alterations in the expression and transcription of genes, thus showing once again the capability of metabolomics to provide information on upstream processes.

## Additional Information

**How to cite this article**: Fernández-Peralbo, M. A. *et al*. Prostate Cancer Patients–Negative Biopsy Controls Discrimination by Untargeted Metabolomics Analysis of Urine by LC-QTOF: Upstream Information on Other Omics. *Sci. Rep.* **6**, 38243; doi: 10.1038/srep38243 (2016).

**Publisher's note:** Springer Nature remains neutral with regard to jurisdictional claims in published maps and institutional affiliations.

## Supplementary Material

Supplementary Information

## Figures and Tables

**Table 1:** Characteristics of the cohort under study.

Variable	PCa (n = 62)	Negative biopsy (n = 42)	*p-*value
Age (years)	71 (64–80)	62.5 (57.8–69)	<0.05
Positive DRE	36 (58.1%)	0	<0.05
*Serum PSA (1) (ng/mL)	11 (6.9–29.75)	4.1 (3.6–5.8)	<0.05
<3	0	3 (7.1%)	
3–10	27 (43.5%)	35 (83.3%)	
>10	35 (56.5%)	4 (9.6%)	
Prostate volumen (mL)	30 (22–40)	27.5 (24–47)	>0.05
N° biopsy (1°)	52 (83.9%)	38 (90.5%)	>0.05
*Serum PSA (2) (ng/mL)	11 (6.9–40.7)	2.3 (1.8–2.7)	<0.05
<3	0	41 (97.6%)	
3–10	28 (45.2%)	1 (2.4%)	
>10	34 (54.8%)	0	
Number of cylinders	12 (12–13)	12 (12–12)	>0.05
Gleason pattern			
6	4 (6.5%)		
7	26 (41.9%)		
≥8	32 (51.6%)		

PSA (1): Serum PSA before the biopsy; PSA (2): Serum PSA on the day of biopsy.

*Data are expressed as median value (range of variability) (Q1-Q3).

**Table 2:** Metabolites tentatively identified by MS/MS with significant different levels in urine from PCa versus that of negative biopsy patients.

Pathway	Compound name	RT (min)	*m/z*	Adduct	Formula	Fragments	*p*-Value	*p*-Value (corrected)	Fold change (Controls vs. PCa)	Regulation in cancer
Lysine degradation	Dimethyllysine***	1.05	175.14409	M + H	C8H18N2O2	84.0815/130.0878	1.01E-06	1.70E-04	1.74	↓
	5-acetamidovalerate***	1.73	160.0968	M + H	C7H13NO3	142.0862/70.0655/98.0623	1.19E-03	2.56E-02	1.51	↓
	Acetyllysine**	1.48	189.1230	M + H	C8H16N2O3	84.0811/126.0914	6.19E-05	2.53E-03	1.33	↓
	Trimethyllysine**	1.05	189.16005	M + H	C9H20N2O2	130.0861/84.0805/60.0806	8.22E-04	1.31E-02	1.28	↓
Histidine metabolism	Imidazole lactate**	1.18	157.0607	M + H	C6H8N2O3	111.0550/83.0610	5.64E-06	3.89E-04	1.46	↓
	Histidine**	1.05	156.07684	M + H	C6H9N3O2	110.0718/83.0625/95.0600	1.69E-06	6.02E-03	1.43	↓
	Methylhistidine**	1.06	170.09247	M + H	C7H11N3O2	124.0864/109.0756/96.0679/83.0600/68.0493	1.64E-03	2.33E-02	1.40	↓
	Acetylhistidine**	1.28	198.0874	M + H	C8H11N3O3	152.0817/110.0717/180.3059	5.84E-03	4.24E-02	1.26	↓
Arginine metabolism	Urea**	1.24	61.0399	M + H	CH4N2O	44.0133	8.39E-03	1.89E-02	1.37	↓
	Acetylarginine**	1.67	215.11621	M-H	C8H16N4O3	173.1032/129.1063	2.35E-03	2.58E-02	1.34	↓
	Acetylcitrulline**	2.52	216.0990	M-H	C8H15N3O4	173.0931/131.0821	3.35E-02	3.75E-02	1.29	↓
	Acetylputrescine**	1.25	131.1178	M + H	C6H14N2O	72.0809/114.0907	5.28E-03	4.19E-02	1.30	↓
	Dimethylarginine**	1.17	203.15036	M + H	C8H18N4O2	70.0653/116.0698/158.1285/88.0874	3.89E-03	3.58E-02	1.25	↓
	Citrulline*	1.34	176.10316	M + H	C6H13N3O3	159.0764/130.0975/70.0651	8.09E-03	4.90E-02	1.23	↓
Tyrosine metabolism	Tyrosine**	2.96	180.0660	M-H	C9H11NO3	119.0503/163.0404/136.0759/93.0318	2.44E-03	2.58E-02	1.34	↓
Tryptophan metabolism	8-methoxykynurenate**	7.79	220.0610	M + H	C11H9NO4	174.0554/116.9734/202.0484	1.39E-02	2.27E-02	1.31	↓
	Kynurenic acid**	7.00	188.0352	M-H	C10H7NO3	144.0454	6.21E-04	1.31E-02	1.30	↓
	Xanthurenic acid*	6.80	206.0450	M + H	C10H7NO4	188.0345/160.0386/132.0459	9.79E-03	1.92E-02	1.22	↓
Taurine metabolism	Sulfoacetate**	1.25	138.9708	M-H	C2H4O5S	94.9810/79.9575	1.56E-03	2.26E-02	1.28	↓
	Isethionate*	1.23	124.9916	M-H	C2H6O4S	79.9572/94.985/106.9797	6.71E-03	4.24E-02	1.19	↓
	Acetyltaurine*	1.56	166.0194	M-H	C4H9NO4S	79.9579/124.0078	1.63E-02	2.27E-02	1.12	↓
Alanine, aspartate and glutamate metabolism	Acetylaspartylglutamic acid*	2.81	303.0833	M-H	C11H16N2O8	96.0077/128.0350	5.21E-03	4.19E-02	1.24	↓
	Acetylaspartate*	1.86	174.0410	M-H	C6H9NO5	88.0403/130.0507/115.0037/58.0296	5.96E-03	4.19E-02	1.19	↓
	2-oxoglutaramate*	1.49	144.03145	M-H	C5H7NO4	126.0202/100.0409/82.0305/72.0454/41.9983	9.85E-04	1.31E-02	1.18	↓
Glutamine and glutamate metabolism	2-pyrrolidone-5-carboxylate**	2.10	128.03542	M-H	C5H7NO3	85.0295/41.0399/110.8772	8.21E-03	4.90E-02	1.25	↓
Purine and pyrimidine metabolism	5-methyldeoxycytidine-5′-phosphate*	2.81	320.0620	M-H	C11H15NO10	110.0245/240.0523	1.88E-02	2.37E-02	1.21	↓
	7-methylguanosine*	5.31	296.100	M-H	C11H15N5O5	164.0574	1.97E-02	2.37E-02	1.15	↓
	7-methylguanine**	2.10	164.0580	M + H	C6H7N5O	149.0497/124.0501	2.64E-02	3.19E-02	−1.30	↑

The compounds are grouped by the metabolism pathways in which they are preferentially involved and sorted by their fold change.

The fold change value ranges are indicated on the table by the following legend:

^***^fold change values from 1.5 to 1.75.

^**^fold change values from 1.25 to 1.5.

^*^fold change values from 1.1 to 1.25.

**Table 3:** Discrimination capability for the target PLS-DA model.

Sensitivity (%)	Specificity (%)	Positive predictive value (%)	Negative predictive value (%)
**Training with 70% of the samples**			
88.4	92.9	95.0	83.9
**External validation with 30% of the samples**			
63.2	78.6	80	61.1
