# Supplementary material for: Prostate Cancer Patients–Negative Biopsy Controls Discrimination by Untargeted Metabolomics Analysis of Urine by LC-QTOF: Upstream Information on Other Omics
Source: Sci Rep. 2016 Dec 2;6:38243. doi: 10.1038/srep38243 (PMC5133625; doi:10.1038/srep38243)
Supplement: Supplementary Information [file srep38243-s1.pdf]

Prostate Cancer Patients – Negative Biopsy Controls Discrimination by Untargeted Metabolomics Analysis of Urine by LC-QTOF: Upstream Information on Other Omics

M.A. Fernández-Peralbo[a,b], E. Gómez-Gómez[b,c], M. Calderón Santiago[a,b], J. Carrasco-Valiente[b,c], J. Ruiz-García[b,c], M.J. Requena-Tapia[b,c], M.D. Luque de Castro[a,b*], F. Priego-Capote[a,b*]

[a]*Department of Analytical Chemistry, Annex Marie Curie Building, Campus of Rabanales, University of Córdoba, E-14071, Córdoba, Spain.*

[b]*Institute of Biomedical Research Maimónides (IMIBIC), Reina Sofía University Hospital, University of Córdoba, E-14004, Córdoba, Spain.*

[c]*Department of Urology (IMIBIC), Reina Sofia University Hospital, University of Córdoba, E-14004, Córdoba, Spain.*

Correspondence: F. Priego Capote and M.D. Luque de Castro, Department of Analytical Chemistry, Annex Marie Curie Building, Campus of Rabanales, 14071 Córdoba, Spain.

E-mails: q72prcaf@uco.es (F. Priego-Capote) and qa1lucam@uco.es (M.D. Luque de Castro).

Tel. and fax number: 34 957 218615.

Supplementary Table 1. Reproducibility values for all identified metabolites, expressed as relative standard deviation (% RSD), calculated prior to and post MSTUS normalization.

Compound Name	Reproducibility (%RSD)	
	Prior MSTUS normalization	Post MSTUS normalization
2-oxoglutaramate	21.65	12.66
2-pyrrolidone-5-carboxylate	25.27	15.90
4-aminohippuric acid	13.38	6.76
4-hydroxy-4-methyl-2-oxoadipate	18.53	10.59
5,6-dihydrouridine	21.07	14.06
5-acetamidovalerate	20.69	8.81
5-methyldeoxycytidine-5´-phosphate	12.51	5.40
7,8-dihydroneopterin	20.46	14.29
7-methylguanine	24.49	10.65
7-methylguanosine	11.72	6.49
8-methoxykynurenate	13.10	9.08
Acetylarginine	16.26	7.21
Acetylaspartate	20.25	10.77
Acetylaspartylglutamic acid	14.06	8.14
Acetylcitrulline	13.12	3.15
Acetylhistidine	26.23	16.00
Acetyllysine	17.16	4.49
Acetylputrescine	33.09	16.26
Acetyltaurine	20.15	13.14
Citrulline	20.98	12.06
Dimethylarginine	25.05	10.37
Dimethylheptanoylcarnitine	13.49	8.52
Dimethyllysine	18.01	10.57
Estrone	7.80	7.44
Histidine	20.46	9.92
Imidazole lactate	28.46	16.00
Isethionate	22.51	13.70
Kynurenic acid	15.36	5.81
Methylhistidine	26.97	14.70
Nicotinic acid	24.50	14.87
Phenylalanine	15.50	5.27
Phosphoric acid	32.19	16.90
Sialyl-N-acetyllactosamine	20.20	15.45
Sulfoacetate	13.58	7.12
Threonine	23.92	13.35
Tiglylcarnitine	12.47	8.37
Trimethyllysine	17.04	13.38
Tyrosine	14.81	5.26
Urea	21.60	14.90
Uric acid	25.43	12.81
Vanillylmandelic acid	16.05	5.89
Xanthurenic acid	14.31	6.50

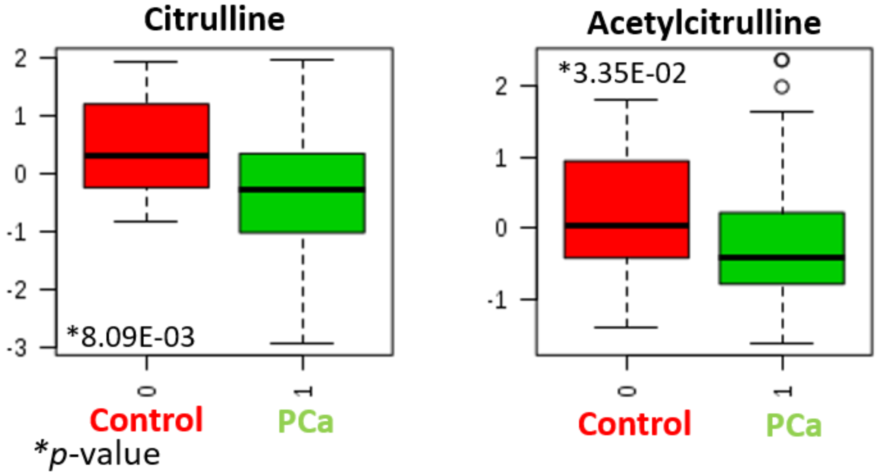

Supplementary Figure 1. Box and whisker plots from citrulline and acetylcitrulline in the two evaluated groups.

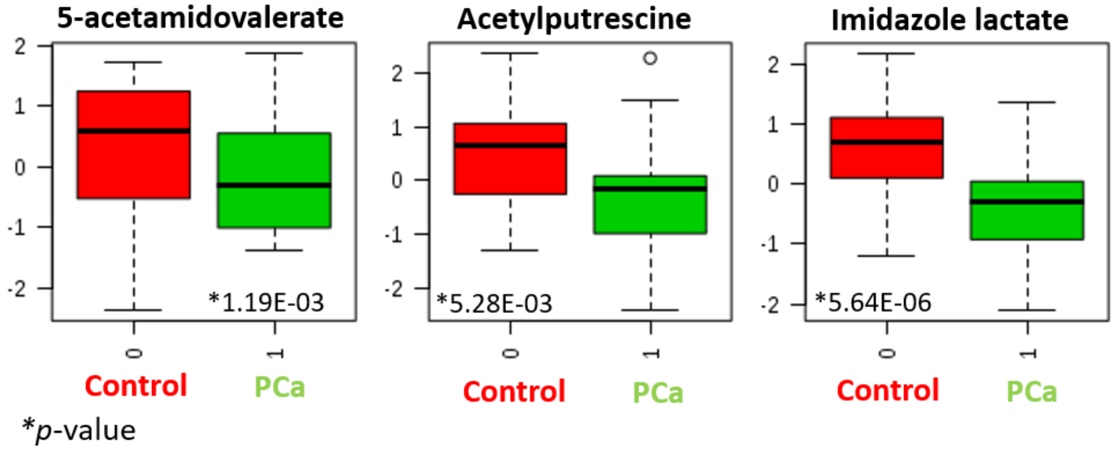

Supplementary Figure 2. Box and whisker plots from 5-acetamidovalerate, acetylputrescine and imidazole lactate in the two evaluated groups.
